# Anti-Atherosclerotic Potential of Free Fatty Acid Receptor 4 (FFAR4)

**DOI:** 10.3390/biomedicines9050467

**Published:** 2021-04-24

**Authors:** Anna Kiepura, Kamila Stachyra, Rafał Olszanecki

**Affiliations:** Chair of Pharmacology, Faculty of Medicine, Jagiellonian University Medical College, 31-531 Krakow, Poland; a.kiepura@uj.edu.pl (A.K.); kamila.stachyra@uj.edu.pl (K.S.)

**Keywords:** free fatty acid receptors, FFAR4, inflammation, atherosclerosis, liver steatosis, apoE-knockout mice, macrophages

## Abstract

Fatty acids (FAs) are considered not only as a basic nutrient, but are also recognized as signaling molecules acting on various types of receptors. The receptors activated by FAs include the family of rhodopsin-like receptors: GPR40 (FFAR1), GPR41 (FFAR3), GPR43 (FFAR2), GPR120 (FFAR4), and several other, less characterized G-protein coupled receptors (GPR84, GPR109A, GPR170, GPR31, GPR132, GPR119, and Olfr78). The ubiquitously distributed FFAR4 can be activated by saturated and unsaturated medium- and long-chain fatty acids (MCFAs and LCFAs), as well as by several synthetic agonists (e.g., TUG-891). The stimulation of FFAR4 using selective synthetic agonists proved to be promising strategy of reduction of inflammatory reactions in various tissues. In this paper, we summarize the evidence showing the mechanisms of the potential beneficial effects of FFAR4 stimulation in atherosclerosis. Based partly on our own results, we also suggest that an important mechanism of such activity may be the modulatory influence of FFAR4 on the phenotype of macrophage involved in atherogenesis.

## 1. Free Fatty Acids and Their Receptors

The progress of civilization has led to a change in lifestyle and nutrition, which clearly contributed to civilization diseases. Excessive consumption of fats, sweets, or alcohol leads to obesity, which increases the risk of diabetes, non-alcoholic fatty liver disease, atherosclerosis, and vascular events. From the mid-20th century, we have witnessed a constant change of eating habits paralleled by technological advances in the food industry that have allowed for the increasing consumption of products containing large amounts of various fats.

Fatty acids are carboxylic acids classified according to the length tail containing saturated or unsaturated bonds into short-chain fatty acids that have fewer than 6 carbon atoms (SCFAs, mainly acetate, propionate, butyrate), medium-chain fatty acids with 6–12 carbons (MCFAs, mainly capric, lauric), and long-chain fatty acids with more than 12 carbons (LCFAs, mainly linoleic, α-linolenic). Long- and medium-chain fatty acids are derived mainly from dietary triglycerides, while short chain fatty acids are produced by gut bacteria in the process of fermentation of indigestible dietary fiber. In animals, fatty acids are also synthesized de novo from carbohydrates mainly in the liver, adipose tissue, and mammary glands [1,2,3]. In tissues, the FAs are present in esters: triglycerides, phospholipids, and cholesteryl esters, however in plasma they are present in a nonesterified, free form (free fatty acids, -FFAs). FFAs are taken up by cells and metabolized in the mitochondria and peroxisomes to carbon dioxide and water, which brings a high ATP yield.

Classically, FFAs have been considered the basic nutrients, but since the 1990s they have been increasingly recognized as signaling molecules by acting on various types of receptors and fatty acid binding proteins (FABPs). Next to the well-characterized intracellular receptors for FFAs such as PPARs [2,4], several types of G protein-coupled membrane receptors have been characterized in recent years [3,5].

G protein-coupled receptors (GPCRs) are seven domains transmembrane receptors that activate various types of the heterotrimeric G proteins. Activation of GPCRs by a ligand leads to the dissociation of the α from βγ subunits of protein G coupled with GPCR and triggers the cascade of well-known intracellular events depending on the type of α subunit, to name just the most important: Gs and Gi/o activating and inhibiting the adenylate cyclase (AC), respectively, and Gq/11 targeting phospholipase C (PLC) and G11/13 affecting the function of large family of Rho small GTPases. Importantly, the βγ subunits of protein G also play an important role in signaling affecting activities of selected isoforms of CA, K+, and Ca++ channels, as well as phosphoinositide-3-kinase isoforms. Both FFAR1 and FFAR4 were reported to recruit β-arrestin 2, however, such action as an alternative signaling pathway has been detailed only for FFAR4 [6,7,8,9,10].

An overview of the current knowledge about FFARs was recently presented in two extensive review papers [2,5]. In this work, we will specifically focus on the function of the FFAR4 receptor and its recently recognized importance in regulating inflammatory mechanisms, in particular on its anti-atherosclerotic potential in the context of an inflammatory theory of atherosclerosis.

The receptors activated by FAs include the family of FFARs of rhodopsin-like receptors: GPR40 (FFAR1), GPR41 (FFAR3), GPR43 (FFAR2), GPR120 (FFAR4), and several other, less characterized GPCRs (GPR84, GPR109A, GPR170, GPR31, GPR132, GPR119, and Olfr78 [1,2,3,5,11]. Major FFAR are activated by distinct classes of FA ligands: the SCFA bind to FFAR3 and FFAR2, the MCFA to FFAR1 and GPR84, and the LCFA to FFAR1 and FFAR4. The overview of ligand preferences and intracellular signaling pathways of the receptors for FFARs are summarized in Table 1.

FFAR1/GPR40 was reported as the receptor activated by MCFAs and LCFAs. This receptor is strongly expressed in the pancreas, L and K cells, immune cells, taste buds, and the CNS. FFAR1 appears to play an important role in the fatty acid–mediated augmentation in insulin secretion and lowering blood glucose in rodent models of type 2 diabetes [2,5]. Upon activation of GPR40, IP3-mediated intracellular Ca 2+ levels increase leading to enhancement of glucose-induced insulin secretion. FFAR1 has been shown to play an important role in lipid metabolism [12]. Recent reports show that GPR40/FFAR1 may be also associated with anxiety- and depression-related behavior regulated by the increment of noradrenaline in the brain [13]. 

FFAR2 and FFAR3 receptors are stimulated by SCFA and are broadly expressed in a variety of human and mouse cells and tissues, e.g., colonic epithelial cells, immune cells (including myeloid cell populations and regulatory T cells), adipocytes, hepatocytes, cardiomyocytes, and neurocytes [2]. These receptors function through Gi and/or Gq signaling and have been shown to modulate a variety of processes: neural signaling, energy metabolism, intestinal cellular homeostasis, immune response, and hormone synthesis. It has been shown that FFAR2 and FFAR3 receptors contribute to the absorption of nutrients, maintenance of the integrity of the intestinal epithelium, and the regulation of intestinal motility. Some studies suggest that activation of FFAR2- and FFAR3-depending signaling may result in alleviation of liver steatosis and benefit in the treatment of obesity [14,15]. Recent years have revealed interesting information about the role of FFAR4 in modulating the activity of cells involved in inflammatory responses. As it has been shown that low-grade inflammation plays a crucial role in many chronic civilization diseases, including diseases of the cardiovascular system, FFAR4 has become a promising target in the search of new therapies.

## 2. The GPR120 (FFAR4)

First described in 2005 [7], the FFAR4 can be activated by both saturated and unsaturated MCFA and LCFA. In humans, there are two FFAR4 splice variants: the long (L) and short (S) isoforms, which differ due to the insertion of 16 amino acids in the third intracellular loop. Some reports suggest that the L variant signals predominantly by the β-arrestin pathway, while S is an equally potent activator of the β-arrestin and Gq signaling pathways [16]. The Gq-independent β-arrestin pathway seems to be particularly important for FFAR4 inhibition of inflammatory stimulation in macrophages (see below).

The expression of FFAR4 is ubiquitous in tissues and cells in the body. Initially, FFAR expression has been demonstrated in human lungs and gastrointestinal tract, as well as in the adrenal gland [7]. Subsequent experiments showed the wide distribution of FFAR4 in different cell types such as: adipocytes, hepatocytes, skeletal muscles, epithelial cells, taste buds, and macrophages [10]. A summary of the reported physiological effects of FFAR4 stimulation is presented in Table 2.

## 3. Pharmacology of FFAR4

An overview of synthetic FFAR4 agonists has been recently published [3]. GW9508 (3-[4-({[3-(phenyloxy)phenyl]methyl}amino)phenyl] propanoic acid), was the first synthetic agonist for FFAR4 and FFAR1 [18]. Next, succeeding NCG21 [4-{4-[2-(Phenyl-pyridin-2-yl-amino)-ethoxy]-phenyl}-butyric acid] and GSK137647A [4-methoxy-N-(2,4,6-trimethylphenyl) benzenesulfonamide] showed 10- and 50-fold higher selectivity to FFAR4 over FFAR1, respectively [19,20]. In 2009 grifolic acid [2,4-dihydroxy-6-methyl-3-[(2E,6E)-3,7,11-trimethyl-2,6,10-dodecatrien-1-yl)]-benzoic acid] was showed to be a partial agonist of FFAR4 [21]. In 2012, Shimpukade et al. reported the ortho-biphenyl ligand 4-{[4-fluoro-4′-methyl(1,1′-biphenyl)-2-yl]methoxy}-benzenepropanoic acid (TUG-891) as a full agonist of the FFAR4, as evidenced by calcium mobilization and β arrestin recruitment assays [8,22]. Hudson et al. compared the TUG-891 activity with endogenous FFAR4 agonist (α-linolenic acid) and demonstrated that the cellular responses for TUG-891 were very similar to those produced by α-linolenic acid [8]. TUG-891 showed 1000-fold selectivity to FFAR4 over FFAR1 in assays on human cells [8,10]. It should be noted that several reports described a reduced selectivity of TUG-891 at mouse FFAR4, nevertheless it remains a valuable tool and a first choice option in the research related to physiology and signaling of FFAR4 [3]. Between 2014 and 2016, other compounds strongly accumulating FFAR4 appeared in basic research: compound A, Metabolex compounds (metabolex 36 and compound B), Astra Zeneca compounds (AZ-423, AZ-670), TUG-1197, and KDT501 [3]. AH-7614, described in 2014 by Sparks et al., is a negative allosteric modulator of FFAR4 [19].

## 4. FFAs and Atherosclerosis

Atherosclerosis is an inflammatory disease that involves many types of cells [23,24]. Dysfunction of endothelial cells, phenotypic changes of vascular smooth muscle cells (VSMC), as well as inflammatory stimulation and monocyte infiltration into the vessel wall all were reported to contribute in concert to atherogenesis [23,25]. Several metabolic diseases like diabetes and obesity—known risk factors of atherosclerosis—can lead to an increased level of FFA, which in turn can aggravate cell activation and inflammation.

FAs might directly activate endothelial cells, which results in the release of numerous chemokines and growth factors, recruitment of circulating monocytes from the blood into the intima, proliferation of VSMC, and synthesis of extracellular matrix components. It was demonstrated that activation of the endothelium with FAs leads to impairment of insulin signaling and nitric oxide production, activation of the renin-angiotensin system, oxidative stress, and dysfunction and apoptosis of endothelial cells [26].

Research by Shen et al. shows that palmitate, the major FFA the circulation may skew VSMC towards the inflammatory phenotype, which is in part mediated by the stimulation of the TLR4/MyD88/NF-κB/NOX1/ROS pathway [27]. Another study has shown that FFA could mediate apoptosis of the VSMCs via the TLR4 pathway and ROS generation [28,29]. Such actions of FFA on VSMC may directly contribute to the progression of atherosclerosis and promote plaque instability. 

High plasma levels of FFA can cause abnormal macrophage activation and their metabolic reprogramming. It has been demonstrated that the FA-dependent TLR4 activation may lead to NLRP3 inflammasome induction and activation of nuclear NF-κB with subsequent generation of ROS and secretion of proinflammatory cytokines. FFA were shown to promote thrombosis by activation of platelets and inhibition of fibrinolysis [30]. Interestingly, there is a growing body of evidence that FFAs acting via FFARs may additionally show important anti-inflammatory effects, hence the stimulation of FFAR, including that FFAR4 might partly counterbalance unfavorable action of FAs in atherosclerosis. It is believed that the stimulation of FFAR4 by DHA may be partly responsible for its beneficial effects on the circulatory system [17].

## 5. FFAR4 and Atherosclerosis

Three types of cells residing in the vascular wall—endothelial cells, macrophages, and vascular smooth muscle cells (VSMC)—play essential role in atherogenesis and regulate plaque morphology. In vitro studies have pointed to many potential mechanisms by which FFAR4 pathway may intervene in cellular changes involved in atherosclerosis. Recently, Jiang et al. demonstrated that GW9508 and TUG- 891 reduced oxidative stress in cultured human aortic endothelial cells (HAECs) via inhibition of the production of ROS and NOX-4, as well as downregulated the expression of VCAM-1 and E-selectin. The latter was associated with a decreased number of THP-1 monocytes attached to HAECs [25]. These results seem to be in agreement with older observations, that DHA (which is a known activator of FFAR4) could inhibit expression of adhesion molecules (ICAM-1, VCAM-1) in TNF-α-induced HUVECs [31].

The protective effects of the FFAR4 pathway on endothelial cells may be related to the activity of Krüppel-like transcription factors (KLFs), a family of zinc finger-containing transcription factors [32]. Although their precise role in endothelial cell function has yet to be elucidated, it was demonstrated that KLF4 was shown to repress arterial inflammation and regulate neointimal formation following vascular injury by inhibiting tumor necrosis factor-α-induced expression of VCAM-1 [33]. Moreover, it was shown that the overexpression of KLF4 in endothelial cells induces the expression of multiple anti-inflammatory and anti-thrombotic factors including endothelial nitric-oxide synthase and thrombomodulin, whereas its knockdown enhances TNF-α-induced VCAM-1 expression [34]. Interestingly, FFAR4 agonists showed the ability to reverse downregulation of KLF4 and induction of adhesion molecules in HAECs [25]. 

The information about FFAR4 role(s) in VSMC is scarce; however, two in vivo studies point to potentially important modulatory role of FFAR4 in VSMC pathology in atherosclerosis. Kamata et al. have shown that EPA inhibits the Tak-1-JNK pathway by activating FFAR-4 in VSMC, which results in the attenuation of the development of abdominal aortic aneurysm [35]. A year later Nakamura et al. showed that stimulation of FFAR4 by EPA caused a decrease of NOX-4 expression and ROS generation in in arterial smooth muscle cells and prevented arterial calcification in klotho mutant mice [36]. Taken together the available data promise FFAR4 as an important VSMC regulator in atherosclerosis; however, clearly more research is required to fully unravel their therapeutic potential.

When it comes to understanding the molecular details of FFAR4′s effects on the pathways of inflammatory cells, most of the research in the field is focused on macrophages. In 2010 Oh et al. showed that activation of FFA by DHA or the synthetic GW9508 agonist significantly increased glucose uptake by GLUT4 translocation through Gαq/11 coupling and activation of PI3 kinase (PI3K). This work also showed, thatω-3 fatty acids can regulate the activity and migration of macrophages through FFAR4 [37]. Moreover, it has been shown that activation of FFAR4 by ω3 fatty acids inhibited macrophage NLRP3 inflammasome activation, production of ROS, and proinflammatory cytokines. The dampening of inflammatory activation of macrophages by FFAR4 depended on stimulation of the β-arrestin pathway. Activation of FFAR4 results in multiple phosphorylations of its carboxy tail, which augments interaction with β-arrestin 2 and recruitment of the TAB-1 protein. This makes the latter unavailable to activate the TAK-1 protein, which is a convergent transducer of TLR4 and TNFα receptor signaling and an activator of NF-kB and MKK4/JNK pathways [2,37,38]. 

In view of the recognized role of macrophages in the development of atherosclerosis, inhibition of their activation by stimulation of FFAR4 may represent an interesting option as an anti-atherogenic strategy. There is ample evidence from animal and human studies that PUFAs may have anti-atherosclerotic potential [39], but only few studies have focused on the role of FFAR4 in this effect. 

Matsumoto et al. showed that EPA suppressed the development of atherosclerotic lesions in apoE-/- and LDL-receptor-/- mice. EPA reduced macrophage accumulation, increased the number of smooth muscle cells, and increased collagen in atherosclerotic plaques. The authors however did not study the role of FFAR4 and attributed the observed effects to the EPA-elicited activation of PPARα receptors in endothelial cells and macrophages [40]. Li et al. studied the involvement of FFAR4 activation by ω3 PUFAs in the process of vascular inflammation and neointimal hyperplasia in mice. The authors used FFAR4-/- strains to study the role of the FFAR4 receptor in vascular dysfunction using acute and chronic thrombosis and vascular remodeling models and found that ω3 PUFAs mitigated vascular inflammation, arterial thrombus formation, and neointimal hyperplasia by interaction with FFAR4 [41]. Interestingly, they showed strong expression of FFAR 4 on perivascular cells (adipocytes and macrophages), which diminished in vascular smooth muscle cells and lymphocytes and dropped to almost no detectable level in platelets and endothelial cells [41], pointing out the main cellular target of the PUFAs’ action.

In a model of the abdominal aortic aneurysm, Kamata et al. pointed to other potentially atheroprotective mechanisms related to FFAR4. The authors showed that EPA via FFAR4 activation reduced the phosphorylation of transforming growth factor beta-activated kinase-1/Map3k7 (Tak-1) and c-Jun NH2-terminal kinase (JNK), as well as downregulated the expression of matrix metalloproteinase-9 (MMP-9) in the media of the aorta; importantly, such effects were mimicked in cultured VSMCs by GW9508 [35]. 

It should be noted that the notion that FFAR4 is responsible only for the beneficial effects of PUFAs is still controversial. In 2017 Shewale et al. conducted an interesting study in vivo to determine the role of leukocyte FFAR4 in ω3 -and ω6 PUFAs-induced atheroprotection. The deletion of FFAR4 in leukocytes, regardless of the type of dietary fat level, had minimal effect on plasma lipid levels, lipoprotein cholesterol distribution, hepatic neutral lipid content, aortic root intimal area, and aortic cholesterol content. The authors concluded that leukocyte FFAR4 expression is neither necessary nor sufficient for atheroprotection in LDLr-/- mice fed ω3 -and ω6 PUFAs-enriched diets [42]. 

**Figure 1:**
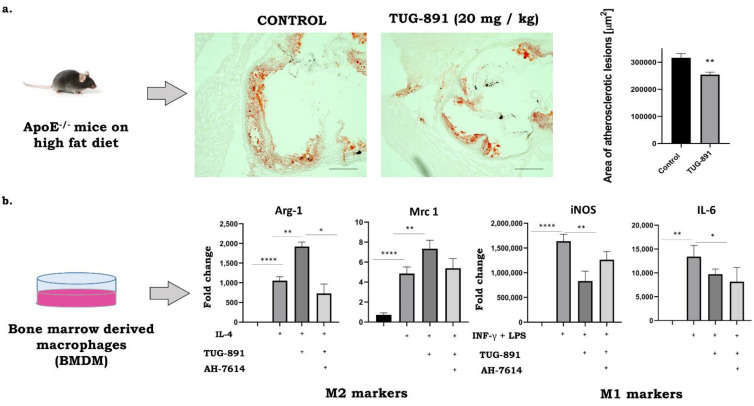
Anti-atherosclerotic activity of TUG-891 in apoE-/- mice. (**a**): The Representative micrographs showing the areas of Oil Red O–stained lesions in control and TUG-891-treated groups (preliminary data of *n* = 5 per group). (**b**): M1/M2 marker expression in bone marrow derived macrophages (BMDM). BMDMs were pre-treated with either 10µM AH-7614 and 10µM TUG-891 for 1 h and then treated with 100 ng/mL LPS + 20 ng/mL INF-γ or 20 ng/mL IL-4 for 24 h and then used in quantitative RT-PCR analyses. (* *p*  <  0.05, ** *p* < 0.01, **** *p*  <  0.0001 as determined by 1-way ANOVA with Tukey’s post-test; *n* = 3–4 per group; unpublished data).

The results from our laboratory indicate that stimulation of FFAR4 by synthetic agonists may have a protective anti-atherosclerotic effect. Suski et al. showed that the activation of the FFAR4 in apoE-/- mice by GW9508 leads to significant decrease of atherosclerosis associated with specific reduction in the number of proinflammatory M1 type macrophages in plaques, as evidenced by immunohistochemical and molecular methods [43]. Interestingly, preliminary data from our laboratory suggest that a much more potent and selective FFAR4 agonist—TUG891—significantly suppresses atherogenesis in apoE-/- mice and such action seems to be associated not only with the decrease of M1 phenotype, but also with the increase of anti-inflammatory M2 macrophages in the plaques and could be reversed by AH7614, an FFAR4 antagonist (Figure 1). Clearly, further research and dedicated experiments are required to fully explore and validate the potential of FFAR4 agonists in regulating the phenotype of macrophages toward an anti-inflammatory M2 activation state as a potential novel pharmacotherapy of atherosclerosis. Whether or not FFAR4 agonists prove to be compounds capable of limiting atherosclerosis by altering the phenotype of cells responsible for maintaining inflammation in the vessel wall remains an attractive hypothesis to be tested.

## 6. Attenuation of Non-Alcoholic Hepatic Steatohepatitis (NASH) as a Possible Mechanism of Anti-Atherosclerotic Action of FFAR4

Substantial evidence from basic research confirms that the dysfunction of liver cells caused by their steatosis may significantly contribute to the maintenance of systemic inflammation and the development of atherosclerosis [44,45]. Additionally, data from clinical trials strongly suggest that non-alcoholic hepatic steatosis and steatohepatitis developed on its background (NASH) are independent risk factors for coronary heart disease [44]. It seems reasonable to assume, that attenuation of liver steatosis by stimulation of FFAR4 might represent an interesting strategy for the prevention of atherosclerosis. Nakamoto et al., showed that DHA supplementation could prevent the development of NASH via FFAR4 signaling [46]. The deletion of FFAR4 exacerbated the inflammatory response in the liver of choline-deficient L-amino acid-defined high-fat (CDAHF) diet-fed mice by increasing the liver content of M1 phenotype macrophages (46). Interestingly, in the study of Raptis et al., the effects of GW9508 were compared with those of Omegaven^®^ (clinical ω3FA-formulation) using a mouse model of hepatic ischemia reperfusion injury (IRI). The protective effect against IRI was similar in both cases and were associated with the shift of macrophage polarization towards M2 [47]. It was also demonstrated that both docosahexaenoic acid and TUG-891 inhibited lipid accumulation in the cultured hepatoma cells, and as such, the effect was attenuated by treatment with AH7614 or transfection of FFAR4 siRNA. The authors showed that activation of FFAR4 in hepatocytes sequentially involves activation of Gq/11 proteins, CaMKK, and AMPK, and suppression of SREBP-1c [48]. Recently the reduction in hepatic inflammation and apoptosis in dietary-induced steatohepatitis upon treatment with FFAR4 agonist were shown in mice fed the High-Fat High-Carbohydrate (HFHC) diet and a diet deficient in methionine and choline MCD [49]. All these data indicate that FFAR4 has the potential to reduce NASH. The answer to the question whether such action may result in reduction of atherosclerosis requires further research.

## 7. Conclusions

**Figure 2:**
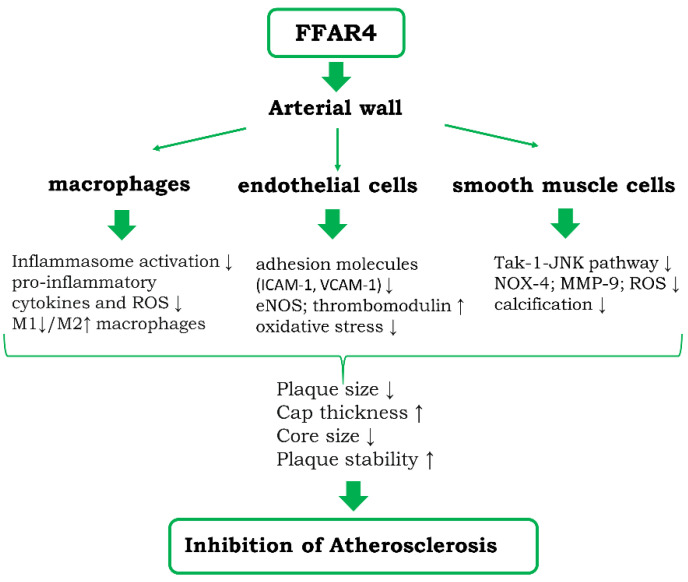
Diagram showing the potential cellular and molecular mechanisms of anti-atherosclerotic actions of FFAR4 within the vessel wall. Potential indirect action of FFAR4 stimulation on atherosclerosis via inhibition of steatosis and steatohepatitis is detailed in the text.

FAs are considered not only as one of the basic nutrients, but also recognized as signaling molecules acting on various types of receptors. The action of FAs through its different receptors appears to offset the unfavorable metabolic and circulatory effects of FAs. The invention of synthetic agonists of individual FA receptors allowed for harnessing FFARs as potential drug targets. Stimulation of FFAR4 on endothelial cells, smooth muscles, and macrophages abrogates the inflammatory stimulation of these cells (Figure 2). The results of FFAR4 activation have been investigated in more details in macrophages, where they appear to be associated with skewing the phenotype of cells to anti-inflammatory M2 type. The stimulation of FFAR4 using selective synthetic agonists proved to be promising strategy of reduction of atherosclerosis and liver steatosis.

## Figures and Tables

**Table 1:** The ligand preference and G-protein-dependent signaling pathways of the free fatty acid receptors (FFARs). AC, adenylate cyclase; Akt (PKB), protein kinase B, DAG, diacylglycerol; IP3, inositol trisphosphate; PI3K, phosphatidylinositol 3-kinase; PKC, protein kinase C; PKD, protein kinase D; PLC, phospholipase C, ω3 PUFAs (ω3 polyunsaturated fatty acids: α-linolenic acid, eicosatrienoic acid, eicosapentenoic acid, docosahexaenoic acid), ω6 PUFAs (ω6 polyunsaturated fatty acids: linoleic acid, γ-linolenic acid, dihomo- γ-linolenic acid, arachidonic acid, docosatetraenoic acid).

Type	Ligands	G-Protein Subunits	Primary Effector	Secondary Effectors, Effects
**FFAR1**	MCFAs	G_s_	AC	cAMP↑
	LCFAs	G_i_	AC	cAMP↓
	ω3 PUFAs	G_q_	PLC	IP3 (Ca^2+^↑)
	ω6 PUFAs			DAG, PKC↑
**FFAR2**	SCFAs	G_i_	PLC	PKC↑, ERK1/2↑
			AC	cAMP↓
		G_q_	PLC	IP3 (Ca^2+^↑)
**FFAR3**	SCFAs	G_i_	AC	cAMP↓
**FFAR4**	LCFAs	G_q_	PI3K	Akt↑
	ω3 PUFAs		PLC	IP3 (Ca^2+^↑)
	ω6 PUFAs			DAG, PKC↑, ERK1/2↑

**Table 2:** A summary of physiological effects of FFAR4 stimulation in various tissues and organs. Based on [3,11,17].

Tissue/Organ	Effects
Hypothalamus	Reduction of food intake, suppression of rewarding effects of high-fat/high-sucrose diet
Taste buds	Taste perception/taste preferences
Adipose tissue	WAT: adipocyte differentiation, lipid accumulation; BAT: stimulation of FGF21 secretion, promotion of browning
Neuroendocrine cells of gastrointestinal tract	Stimulation of CCK and GLP-1 secretion
Delta cells of pancreas	Stimulation of somatostatin secretion
Bones	Decrease of osteoclastic bone resorption, stimulation of osteoblastic bone formation
macrophages	Attenuation of inflammatory macrophage activity

## Abbreviations

AMP	Adenosine monophosphate
AMPK	5′ AMP-activated protein kinase
ApoE-/-	apoE-knockout mine
ATP	Adenosine triphosphate
BAT	brown adipose tissue
Ca^2+^	intracellular calcium
CaMKK	Ca^2+^/calmodulin-dependent protein kinase
CVD	Cardiovascular disease
DHA	Docosahexaenoic acid
ED	Endothelial dysfunction
eNOS	Endothelial NOS
EPA	Eicosapentaenoic acid
ER	Endoplasmic reticulum
ERK ½	extracellular signal-regulated kinases ½
FFA	Free fatty acid
FFA1	free fatty acid receptor 1
FFA2	free fatty acid receptor 2
FFA3	free fatty acid receptor 3
FFA4	free fatty acid receptor 4
GLUT4	Glucose transporter type 4
GPCR	G protein-coupled receptors
GPR 120 agonist III	3-(4-((4-Fluoro-4′-methyl-(1,1′-biphenyl)-2-yl)methoxy)-phenyl)propanoic acid
GPR 120	G-protein coupled receptor 120
GSK137647A	4-Methoxy-N-(2,4,6-trimethylphenyl)-benzenesulfonamide, N-Mesityl-4-methoxybenzenesulfonamide
GW9508	4-(3-Phenoxybenzylamino)phenylpropionic acid
HFHC	High-fat–high-cholesterol
ICAM-1	Intercellular Adhesion Molecule 1
JNK	c-Jun N-terminal kinases
KLF2	Krüppel-like factor 2
KLF4	Krüppel-like factor 4
KLFs	Krüppel-like family of transcription factors
LCFA	Long-chain fatty acids
M1-type	classically activated macrophage
M2-type	alternatively activated macrophage
MAP3K7	Mitogen-activated protein kinase kinase kinase 7
MCD	Methionine and choline deficient
MCFA	medium-chain fatty acids
MMP-9	Matrix metallopeptidase 9
n-3 PUFA	polyunsaturated fatty acids
NADPH	Nicotinamide adenine dinucleotide phosphate
NASH	Nonalcoholic steatohepatitis
NCG21	4-{4-[2-(Phenyl-pyridin-2-yl-amino)-ethoxy]-phenyl}-butyric acid
NF-κB	Nuclear factor kappa B
NLRP3	NLR family pyrin domain containing 3
NOX-4	NADPH oxidase 4
PI3K	Phosphoinositide 3-kinase
PRARα	Peroxisome proliferator-activated receptor alpha
PUFA	Polyunsaturated fatty acid
ROS	Reactive oxidative stress
RT-PCR	reverse-transcription polymerase chain reaction
SCFA	Short-chain fatty acids
SFAs	Saturated fatty acids
siRNA	Small interfering RNA
SMC	smooth muscle cell
SREBP-1c	sterol regulatory element-binding protein 1c
T2DM	type 2 diabetes mellitus
THP-1	human monocytic cell line
TLR4	Toll-like receptor 4
TNF-α	tumor necrosis factor α
VCAM-1	Vascular cell adhesion protein 1
VSMCs	Vascular smooth muscle cells
WAT	white adipose tissue
